# A matter of taste: Spatial and ontogenetic variations on the trophic ecology of the tiger shark at the Galapagos Marine Reserve

**DOI:** 10.1371/journal.pone.0222754

**Published:** 2019-09-20

**Authors:** Pelayo Salinas-de-León, Denisse Fierro-Arcos, Jennifer Suarez-Moncada, Alberto Proaño, Jacob Guachisaca-Salinas, Diego Páez-Rosas

**Affiliations:** 1 Charles Darwin Research Station, Charles Darwin Foundation, Puerto Ayora, Galapagos Islands, Ecuador; 2 Pristine Seas, National Geographic Society, Washington, DC, United States of America; 3 Galapagos National Park, Puerto Ayora, Galapagos Islands, Ecuador; 4 Facultad de Ciencias Naturales, Universidad de Guayaquil, Guayaquil, Ecuador; 5 Universidad San Francisco de Quito, Galapagos Science Center, Isla San Cristóbal, Galapagos Islands, Ecuador; Institut de recherche pour le developpement, FRANCE

## Abstract

Sharks are top predators across ocean food webs and have a major ecological role in marine ecosystems. Investigating the trophic ecology of this group of species is thus essential to understand ecosystem functioning and inform specific management actions aimed at shark conservation. The Galapagos Islands represent one of the last ocean wildernesses, where populations of sharks and other top marine predators come close to a pristine status. Here we provide the first study on the trophic ecology of the tiger shark (*Galeocerdo cuvier*) within the Galapagos Marine Reserve (GMR), using a combination of stable isotope analysis, satellite tracking, and passive acoustic telemetry to investigate ontogenetic and spatial variations at two regions. The mean estimated δ^13^C and δ^15^N at Isabela island (western region) were -13.9 ± 0.5‰ and 13.7 ± 0.7‰; and for Santa Cruz island (central region) were -13.8 ± 0.3‰ and 13.4 ± 0.7‰, respectively. Green sea turtles (*Chelonia mydas*) were the main prey item for large tiger sharks (>280 cm TL), while smaller sharks mainly fed on squid and pelagic fish. Tiger sharks exhibited a high degree of philopatry around green sea-turtle nesting areas, with the majority of sharks detected around green sea-turtle nesting areas for at least 10 months after their capture date, and some individuals were even present during the entire three-year study period. Although we did not report statistically significant differences between the two regions, isotopic and electronic tagging data suggest that tiger sharks in the Galapagos could be segregated into specific populations separated by geographical scales of <100 km. The high productivity of the archipelago, along with the protection from industrial fishing granted by the GMR, result in abundant and predictable sources of prey. This high food abundance, combined with the presence of suitable habitats throughout the tiger shark life cycle, might result in a reduction of migratory behaviours when compared to movement patterns of tiger sharks in other ocean basins. Additional studies using genetic tools could provide further evidence on the presence of separate management units, as it has been recently revealed for other shark species inhabiting the GMR.

## Introduction

Most sharks are apex predators across ocean food webs and have a major ecological role in coastal and open water systems [1,2]. The influence of sharks on prey populations can occur via direct predation (also known as ‘lethal effects’); and via behavioural mediated indirect effects (i.e., ‘risk effects’), in which predation risk triggers anti-predator responses that can result in reduced foraging rates, and in turn affect reproductive output and recruitment [3]. Despite the importance of sharks in maintaining healthy marine ecosystems, many shark populations have declined sharply globally, mainly due to overfishing [4,5]. This decline has resulted in the release of mesopredator prey populations from predatory control, and consequently, a sharp decline in the abundance of smaller prey species via trophic cascades [6–8]. To better understand the impacts of predator removal on marine ecosystems, it is thus essential to improve our understanding of the ecology of apex predators, in particular their trophic ecology.

The tiger shark (*Galeocerdo cuvier*) is one of the largest ocean predators, reaching up to 550 cm total length (TL) [9]. They have a worldwide distribution across tropical and warm-temperate coastal and pelagic waters [10]. Most of the information available on their diet has been obtained from stomach content analysis, which revealed they are generalist predators that consume a wide range of invertebrate and vertebrate prey items [11–13]. These prey items include reptiles, birds, marine mammals, teleost, elasmobranchs, crustaceans, molluscs, and even human derived food [10,12,13]. Tiger sharks are also known to trigger ‘risk effects’ on a number of prey including dugongs (*Dugong dugon*) [14], bottlenose dolphins (*Tursiops truncatus*) [15] and green sea turtles (*Chelonia mydas*) [16]. Most tiger diet studies have also revealed ontogenetic dietary shifts, with larger sharks feeding on larger prey and a wider range of species [12,13]. For example, Simpfendorfer et al. [12] reported that larger tiger sharks in Australia fed on turtles, birds, and elasmobranchs, while smaller sharks fed predominantly on sea snakes and teleosts.

Spatial variations in the diet of tiger sharks have also been documented across several ocean basins, in many cases taking advantage of seasonally abundant food sources. For example, sea snakes are one of the most common prey items of tiger sharks in northeastern Australia [12] and New Caledonia [11], whereas sea turtles and dugongs are an important prey item in Shark Bay, Western Australia [14,17]. In the northwestern Hawaiian Islands, seabirds are the most important prey items [18]; while tiger sharks around the main Hawaiian islands display a broader diet composition [13,19]. A limited number of studies are available from other geographical areas, such as the Indian [20], Atlantic [21] or Eastern Pacific [22] ocean basins. In the Tropical Eastern Pacific, diet studies for this species have been limited to the analysis of stomach contents from just six individuals from the coast of mainland Ecuador [22]. Tiger sharks in the Galapagos Marine Reserve (GMR) display a strong philopatric behaviour around green sea-turtle nesting beaches, and it has been hypothesized that this site fidelity could be related to the local availability of food [23]. However, to date no study has addressed the trophic ecology of this top predatory shark at this UNESCO Marine World Heritage Site.

Although stomach content analyses provide the most accurate information on food items ingested, this technique has limitations and biases, including the snapshot nature of only recently consumed prey and the differential digestion rates of prey, which must be considered when interpreting results [1]. The stable carbon and nitrogen isotope analysis provides a reliable and non-lethal alternative to study the trophic relationships among sharks and other organisms within the ecosystems they inhabit [24–26]. This analysis is based on the fact that ratios of nitrogen (^14^N/^15^N) and carbon (^12^C/^13^C) isotopes in the tissue of predators provide information about the prey they consumed, the type of habitat they utilise (coastal/oceanic, using δ^13^C), as well as their trophic level and breadth (using δ^15^N) [27,28]. The isotopic information present in the tissues of a predator can be used as a natural chemical tracer of ecological processes, allowing researchers to identify energy flows and characterize the primary production sources that sustain the ecosystem inhabited by a predator [29,30].

Non-invasive methods (e.g., stable isotopes analysis) are also a valuable approach in areas where fisheries-derived data might not be available, such as within Marine Protected Areas where the capture or handling of sharks might not be permitted. Within the Tropical Eastern Pacific, the Galapagos Islands remain as one of the best preserved oceanic archipelagos and harbours a great abundance of sharks and other top predators [31]. The Galapagos also host one of the most important rookeries for green turtles in the eastern Pacific Ocean, representing more than 40% of the green turtles in the region [32]. Highly isolated or heavily protected areas where marine ecosystems remain close to their pristine state, present a unique lens to understand the feeding behaviour of apex predatory sharks with limited human interference [33,34]. This is the first study to evaluate spatial and ontogenetic variations on the feeding behaviour of tiger sharks within the GMR and the Eastern Pacific.

## Materials and methods

### Ethics statement

This research was approved by the Galapagos National Park Directorate (GNPD) as part of the research permits granted to Dr. Pelayo Salinas-de-León from the Charles Darwin Foundation (Permits: PC-40-14, PC-17-15, PC-28-16, PC-27-17, and PC-46-18), and Dr. Diego Páez-Rosas from the University San Francisco de Quito (Permits: PC-38-16, PC-24-17, and PC-69-18). The methods described here were reviewed and approved by the GNPD committee responsible for assessing animal welfare in research activities.

### Study site

**Fig 1:**
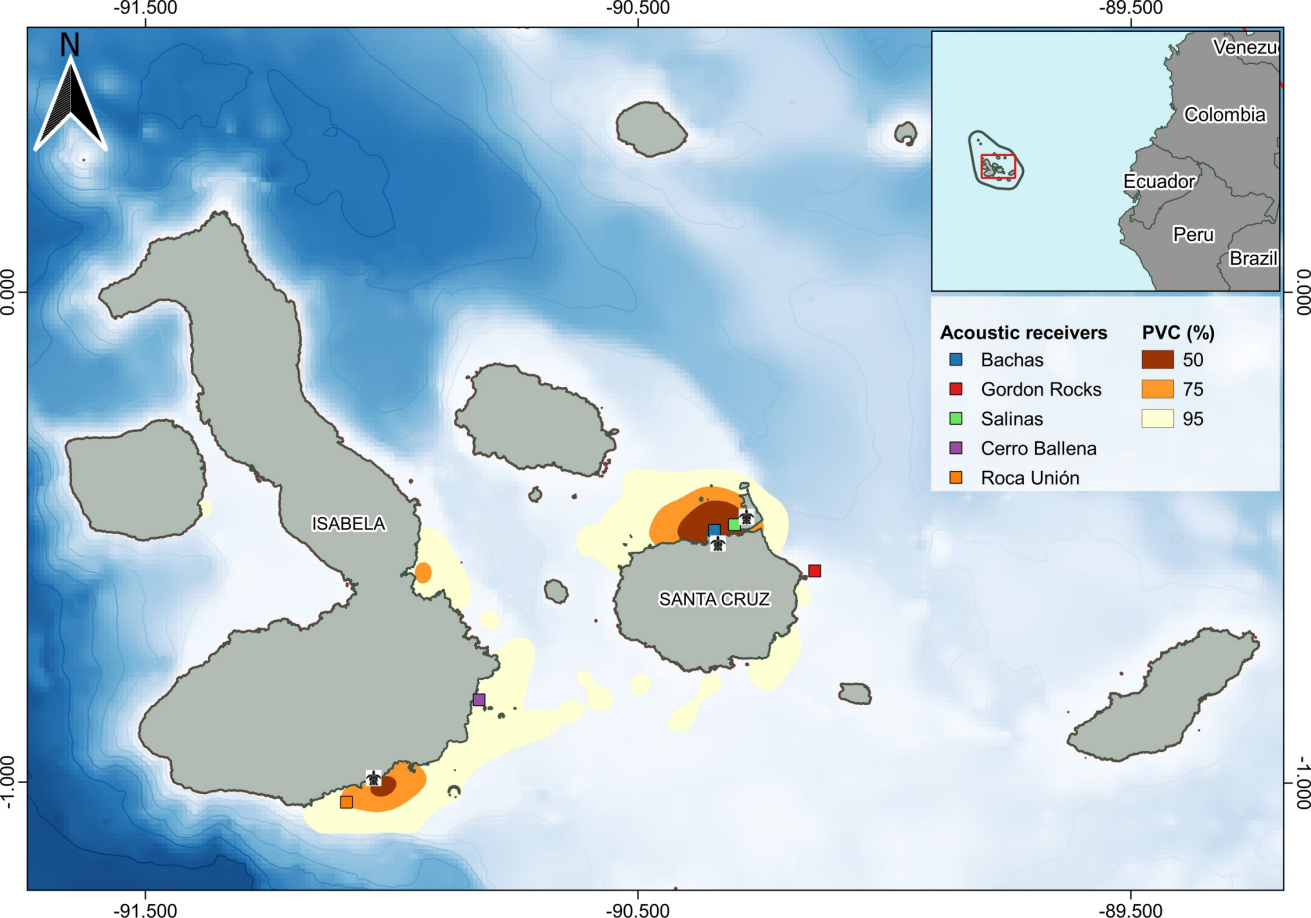
Kernel density estimates (KDEs) of satellite-tagged (n = 21) tiger sharks (*Galeocerdo cuvier*) locations within the Galapagos Marine Reserve. The 50% Percent volume contours (PVCs) in red represent areas of concentrated use, PVC 95% (yellow) represent activity spaces, and PVC 75% (orange) represent intermediate use areas. Sea turtle images show the location of green-turtle nesting beaches, and the coloured squares represent the location of passive acoustic receivers. Bathymetry contours shown at 500 m depth intervals (CRS: WGS84).

The Galapagos Islands are located approximately 1,000 km from mainland Ecuador, in the Tropical Eastern Pacific ecoregion (Spalding et al. 2007) (Fig 1). The Galapagos Marine Reserve (GMR), established in 1998, banned industrial fishing and the capture of sharks and other megafauna in an area of approximately 138,000 km^2^ around the archipelago. Thanks to this protection, the GMR represents one of the last ocean wildernesses where sharks and other trophic groups are close to their pristine status [31,32,35,36].

### Feeding behaviour

#### Tissue sample collection

Muscle tissue samples were collected from free swimming tiger sharks captured around the south of Isabela and the north of Santa Cruz islands between January 2014–2017 as part of a multi-institutional research project focused on the conservation of sharks within the Galapagos Marine Reserve (Table 1). Sharks were captured at these locations due to sightings and records of possible interactions between tiger sharks and green sea turtles (*Chelonia mydas*) that nest around these islands [23].

**Table 1:** Summary of electronic tags deployed and tissue samples collected for isotopic analysis on tiger sharks (*Galeocerdo cuvier*) at Santa Cruz and Isabela Islands within the Galapagos Marine Reserve. ^+^Individual recorded over 10 months after tagging date. ^#^Individual still present at the end of the study in October 2017. () Failed tag. ^ Individual tagged at Isabela detected on the Santa Cruz receivers. * Data obtained from Acuña-Marrero et al. [23].

Shark ID	Sex	TL (cm)	Date capture	Acoustic tag ID (n = 26)	Satellite tag ID (n = 20)	Stable Isotope (n = 29)
**Isabela Island**						
GC230714-1	F	140	23/07/14	24894		Y
GC230714-2	M	224	23/07/14	24893	139131*	Y
GC240714-1	F	234	24/07/14	24890^	139129*	
GC230714-2	F	171	24/07/14	24891	139137*	
GC230714-3	F	260	24/07/14		139128*	
GC071014-1	M	180	7/10/14	24892	(139135*)	Y
GC071014-1	M	180	7/10/14	24895	(139136*)	Y
GC210215-1	M	206	21/02/15	52983^	139130*	Y
GC210215-2	M	202	21/02/15	52980	139132*	Y
GC220215-1	F	378	22/02/15		139133*	Y
GC220215-2	F	282	22/02/15	32391	139134*	Y
GC220215-3	M	324	22/02/15	24888	103758*	Y
GC230215-1	M	286	23/02/15	24887	53957*	Y
GC230215-2	M	242	23/02/15	24889^	53801*	Y
**Santa Cruz Island**						
GC300114-1	F	274	30/01/14	27483^+,#^	132356*	Y
GC300114-2	F	251	30/01/14	27482	121416*	Y
GC300114-3	F	248	30/01/14	(27480)	132358*	Y
GC300114-4	F	383	30/01/14	27484^+,#^	121417*	Y
GC110615-1	F	225	11/06/15	52979^+,#^		Y
GC110615-2	F	240	11/06/15	24886^#^	53996*	Y
GC130716-1	F	337	13/07/16	25726^+,#^	157572	Y
GC130716-2	F	220	13/07/16	25722^+,#^	157571	Y
GC100816-1	M	330	10/08/16	22979^+,#^	157570	Y
GC100816-2	F	227	10/08/16	22975^+,#^	157573	Y
GC190117-1	M	340	19/01/17	22953^#^		Y
GC190117-1	F	305	19/01/17	22982^+,#^		Y
GC190117-1	M	260	19/01/17	22950		Y
GC200117-1	F	395	20/01/17	(22978)		Y
GC200117-2	F	370	20/01/17	22946^+,#^	157574	Y
GC200117-3	F	390	20/01/17	(22954)		Y
GC200817-1	M	370	20/08/17	Not used		Y
GC200817-2	M	413	20/08/17	Not used		Y

Tiger sharks were captured following the methodology described by Acuña-Marrero et al. [23]. All sharks were attracted to a 7.5 m long fishing skiff using fish burley and captured using handlines baited with yellow-fin tuna (*Thunnus albacares*). Captured sharks were secured alongside the vessel and inverted to induce tonic immobility [37]. Each shark was sexed and measured to the nearest cm (total length, TL). Based on previous studies, it was decided to classify individual samples in two categories: small (< 280 cm TL) and large sharks (> 280 cm TL) to reduce variability when comparing among individuals [9,13] (Table 1).

In order to understand the trophic relationship between tiger sharks and green turtles (*C*. *mydas*), we also obtained skin samples from nesting green sea turtles at Quinta Playa in Isabela (n = 30), and at Bachas beach in Santa Cruz (n = 30). Green turtle skin samples were taken by Galapagos National Park Directorate rangers while conducting their annual nesting monitoring program during the 2016 and 2017 nesting seasons (Fig 1) from biopsy punches in the trailing margin of a rear flipper. Only adult females were sampled, as they were the most abundant category in the nesting area.

#### Sample processing

Shark muscle and green turtle skin samples were rinsed with deionized water to eliminate residues that could alter their isotopic signature. Samples were placed in glass vials, which were previously treated for 24 h with a chromic acid mixture prepared from sulfuric acid and potassium dichromate. Samples were then dried in a desiccator at 80°C for 12 h to remove all moisture. A microwave-assisted extraction protocol (MAE) was applied (Microwave oven model: 1000-W MARS 5x, CEM, Matthews, USA) using 25 ml of a 1:1 chloroform/methanol solution [38] and dried again. Samples were homogenized with an agate mortar to obtain a very fine powder, of which ∼1 mg was weighed by means of an analytical microbalance with a precision of 0.001 mg and transferred into a tin capsule for isotopic analysis.

δ^13^C and δ^15^N stable isotope ratios were determined by a PDZ Europa 20–20 continuous-flow isotope-ratio mass spectrometer (Sercon Ltd., Cheshire, UK) at the Stable Isotope Facility of the University of California at Davis (CA, USA). Results, expressed in parts per thousand (‰), were obtained using the following equation:

$$\delta^{13}C\;or\;\delta^{15}N=1000*\left(\frac{R_{sample}}{R_{standard}}-1\right),$$

where R_sample_ and R_standard_ are the ^13^C/^12^C or ^15^N/^14^N ratios of the sample and the standard, respectively. The standards used were Pee Dee Belemnite (PDB) for δ^13^C and atmospheric N_2_ for δ^15^N. Within-run analytical precision was estimated via analysis of two proteinaceous internal reference materials, which was ± 0.2‰ for both δ^13^C and δ^15^N values. We also measured the weight percentage of carbon and nitrogen concentration of each sample and used the C/N ratio as a proxy of lipid content [39].

#### Isotopic data analysis

Data were tested for normality and homoscedasticity using the Shapiro-Wilk and Levene test, respectively. Parametric tests were used to determine if there were differences in δ^13^C and δ^15^N values among the groups. Differences were reported to be statistically significant when *P* < 0.05. All statistical analyses were performed in Statistica 8.0.

The percentage contributions of different prey items to the diet of tiger sharks were evaluated with a Bayesian Mixing Model based on isotopic values implemented in the R package ‘SIMMR’ [40]. This model estimates the probability distribution of the contribution of n sources (i.e., prey items) to a mixture, also evaluating the uncertainty associated with the isotopic values of the prey sources and predator [40]. We used published data of isotopic values for squids (*Dosidicus gigas*, *Sthenoteuthis oualaniensis*, *Ommastrephes bartramii*) [35,41], pelagic fish (*Selar crumenophthalmus*, *Sardinops sagax*, *Anchoa sp*., *Thunnus albacares*, *Katsuwonus pelamis*, *Acanthocybium solandri*) [26,35,42], demersal fish (*Pontinus clemensi*, *Paralabrax albomaculatus*, *Semicossyphus darwini*) [35,42], and sea lions (*Zalophus wollebaeki*) [43]. The isotopic data of green turtles (*C*. *mydas*) collected for this study was also used as a prey source for this model. The mixing models require assumptions about the trophic enrichment factor (TEF) between consumers and their sources [44]. The isotopic values for the diet items need to be adjusted based on the TEF to accurately assess the contribution of each prey item to the consumer's diet [30,45]. Controlled feeding studies have indicated that TEFs are species and tissue specific [46,47], and TEFs can be based on values reported in the literature [44]. Unfortunately, there are few experimental studies examining TEFs for elasmobranchs, and even fewer that have evaluated tiger sharks specifically. In this work, we assumed a TEF for δ^15^N of 2.3 ± 0.8‰ and 3.2 ‰ ± 0.8‰ for δ^13^C, following [28] who calculated these values for muscle of sharks in the Eastern Tropical Pacific.

The Bayesian SIBER (Stable Isotope Bayesian Ellipses in R) package method provides a measure of the isotopic resource use area at the population level, and it was used to define the isotopic niche space among age categories, sites and sex. Bayesian Approach Models that use δ^13^C and δ^15^N values have provided a better understanding of the trophic behaviour of marine predators. These models can be adjusted to rule out an imprecise hypothesis, which allows the uncertainty involved in the contribution of the energy sources to be described in probabilistic terms [48]. This method is based on the two-dimensional isotopic space of δ^13^C/δ^15^N and assessed using Bayesian analysis of standard ellipses, which allows for an unbiased estimate of relative isotopic niche based on fewer samples than those needed in Euclidean approaches (e.g., convex hulls). Therefore, our estimates of isotopic niche should not be impacted by sample sizes, although this could result in greater uncertainty and therefore credible intervals [49]. Monte Carlo simulations were used to correct the bivariate ellipses (δ^13^C and δ^15^N) surrounding data points within the 95% confidence interval for the distributions of both stable isotopes [48]. These corrected standard ellipse areas (SEAc) represent the isotopic niche width and overlap parameters. Furthermore, we calculated the magnitude of the isotopic overlap among the groups based on 100,000 posterior draws of the SEAc parameters.

### Movement patterns and site fidelity

All individuals captured and used for the diet study were fitted with at least one type of electronic tag to study their movements and site fidelity around green sea turtle nesting areas (Table 1). Satellite SPOT tags (Wildlife Computers Ltd., Washington, USA) were attached to the first dorsal fin and acoustic V-16-6x transmitters (VEMCO Ltd., Nova Scotia, Canada) were surgically implanted into the intraperitoneal cavity of the shark following Meyer et al. [50].

#### Satellite data analysis

A total of 21 tiger sharks were tagged with SPOT satellite transmitters between January 2014 and November 2017 (Table 1). Data for 16 of these individuals was obtained from [23]. The SPOT satellite tags transmit a short radio signal to polar-orbiting NOAA satellites when the tags are above the surface of the water. These transmissions do not provide the exact position of the tag, instead its location is estimated by the Argos Data Collection and Location Service from the Doppler shift in the frequency of transmissions received by a satellite as it moves towards and away from the tag on a single overpass [51]. For a detailed description of the functioning of satellite tags see Hammerschlag et al. [52].

Due to the inherent errors and irregularity in the frequency of Argos estimated locations, it is not appropriate to use the raw location estimates to perform temporal and between-individual comparisons. Instead, data was normalized by applying a Bayesian state-space model (SSM), which calculates the most likely geolocation for each raw location by taking into consideration the error associated to its location class, mean turning angle, and autocorrelation in the speed and direction of the tagged animal [53]. SSM also provides location estimates at regular time intervals, which results in a more complete track for each individual tagged.

To ensure the results of the SSM were more robust, we excluded any incomplete and/or duplicate entries from the raw dataset as well as any points estimated to be over 5 km inland as this was the maximum error radius reported for the Argos data [52]. Additionally, individual tracks were partitioned into smaller segments if gaps of over seven days were present [23]; however, if the resulted partitions contained less than 10 raw location points, these subsets were excluded from further analysis. Finally, data was split into years, as there were data gaps of over two months between years, which may have resulted in imprecise model estimations [54].

The SSM was performed using the ‘bsam’ package [55] in R [56]. The hierarchical difference correlated random walk switching (hDCRWS) model was used to simultaneously estimate the location for multiple individuals. This model was chosen as it takes into account the estimated positions and their behavioral mode, resident or migratory, at both the individual and group level [55]. Estimating patterns of movement across individuals results in more accurate track estimates as all available individuals are used to determine when changes in behavior occur [57]. A 12-hour interval was used in the SSM because 79.74% of the time gaps between positions were under 12 hours [23,58].

Spatial kernel density estimates (KDEs) were used to identify areas of concentrated use. KDEs were calculated using projected (EPSG: 32715) SSM results located within the boundaries of the GMR (91.68% of data points) using the ‘spatialEco’ package in R [59]. The bandwidth or kernel size has a major influence on the results of the KDE [60], thus the bandwidth for this analysis was calculated using Silverman’s rule of thumb:

$$h=1.06*\sigma *n^{0.2},$$

where *h* is the bandwidth, *σ* is the standard distance deviation (SDD) of the estimated location and *n* is the number of estimated locations. The SDD is similar to the standard deviation as it measures the variance in the spatial data around its mean center. The SDD was calculated using the ‘aspace’ R package [61] and used as input for the KDE calculation. A mask was applied to the resulting KDEs to filter out any points located on land. Finally, percent-volume contours (PVCs) were calculated using the filtered KDEs. The 50% PVC represented the core range (CR), while the 95% PVC indicated the activity space (AS) of tiger sharks within the GMR, and the 75% PVC represents an intermediate use area [23]. PVCs were calculated using the *raster*.*vol* function of the ‘spatialEco’ package [59]. The final layer produced was exported as a tif raster file into QGIS 3 [62] to produce the final map. The script developed to perform these analyses is available for download: https://github.com/lidefi87/FCD_R_code/blob/master/AnalysingArgosData_v2.R.

#### Acoustic data analysis

Five acoustic receivers (VR2W, Vemco Ltd., Nova Scotia, Canada) were deployed to investigate the site fidelity of tiger sharks around green sea turtle nesting beaches (Fig 1). For a detailed description on passive acoustic tracking refer to Meyer et al. [50]. Two receivers were located around green sea turtle nesting beaches to the north of Santa Cruz Island (Bachas and Salinas), and two receivers were located to the south of Isabela Island (Quinta Playa and Cerro Ballena) following the design by [23] (Fig 1). An additional receiver was located at Gordon Rocks located to the east of Santa Cruz Island and over 20 km away from Salinas beach (Santa Cruz Island) to serve as a control. All receivers were deployed between October 2014 and October 2017.

Data was pre-processed by removing single tag detections that could be caused by signal collision or noise. Since Acuña-Marrero et al. [23] established a residency index and a diel cycle of use around nesting areas during 2014–2015, we produced an abacus plots of daily tiger shark detections to examine site fidelity patterns over the entire duration of this study (January 2014—October 2017).

## Results

### Isotopic comparison between sites, ages and sexes

**Fig 2:**
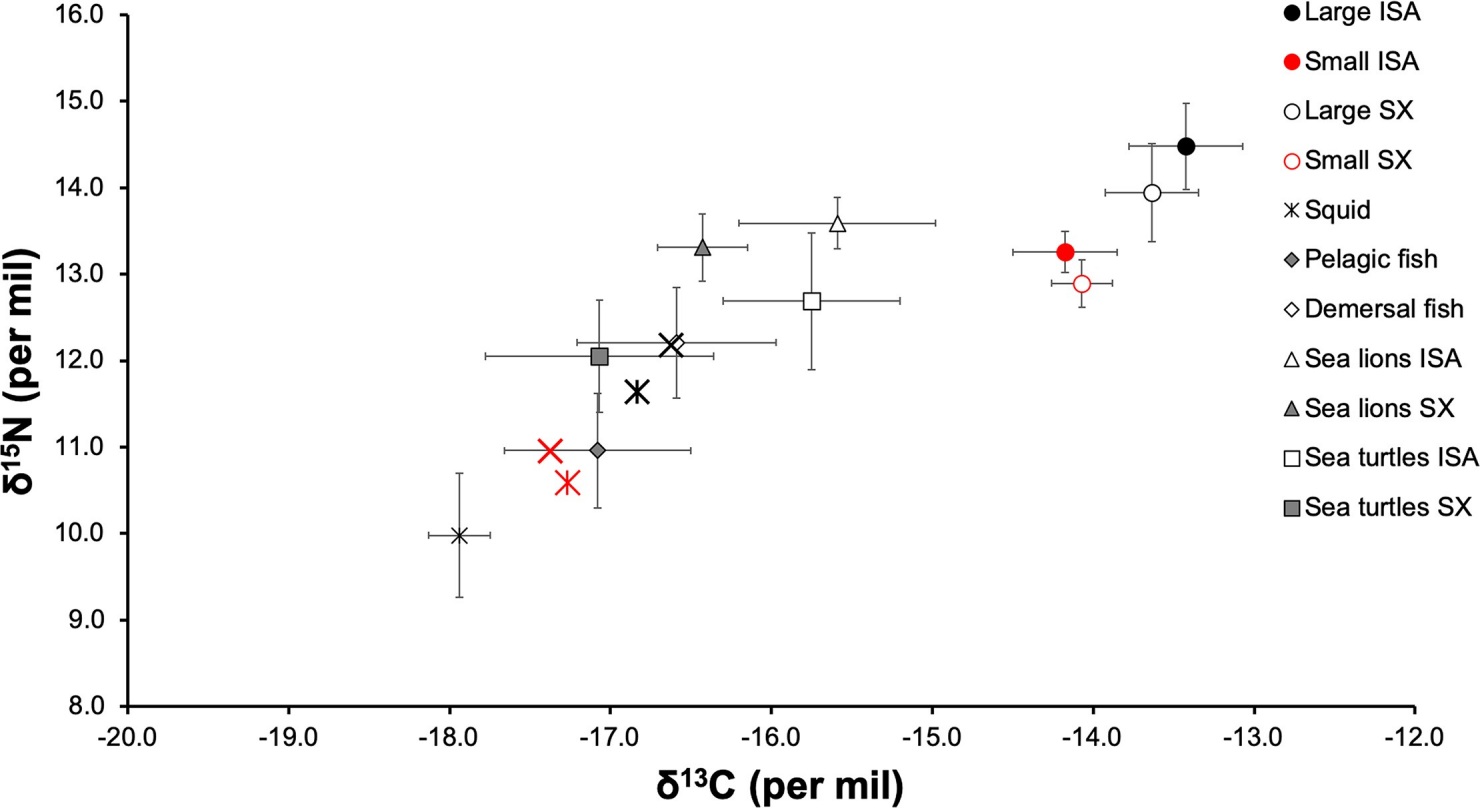
Values of δ^13^C and δ^15^N (mean ± SD in ‰) in large and small tiger sharks (*G*. *cuvier*) sampled at two locations within the Galapagos Marine Reserve, together with isotopic values for different groups of potential prey that make up the trophic web of this species: squids (asterisk), pelagic and demersal fish (diamonds), sea lions from Isabela and Santa Cruz rookeries (triangles), and sea turtles from Isabela and Santa Cruz nesting areas (squares). For tiger sharks: Large from Isabela (Black X), small from Isabela (red *), large from Santa Cruz (black *), small from Santa Cruz (red *). Values were adjusted with the Trophic Enrichment Factor (TEF) proposed by Malpica et al. [47].

The mean estimated δ^13^C and δ^15^N values in the muscle tissue of *Galeocerdo cuvier* at Isabela were -13.9 ± 0.5‰ and 13.7± 0.7‰, respectively; and for Santa Cruz were -13.8 ± 0.3‰ and 13.5 ± 0.7‰, respectively. The C/N ratios of the samples ranged from 2.8 to 3.1, and thus were within the theoretical range established for the assimilation of protein from a predator’s diet [63]. There were no significant differences among islands in δ^13^C and δ^15^N values (Mann–Whitney U test, p = 0.70 and 0.31 respectively), nor between sexes (Mann–Whitney U test, p = 0.65 and 0.68 respectively). The δ^13^C and δ^15^N values, in contrast, were significantly different between size categories (Paired t-test, p < 0.01 and 0.01 respectively) (Table 2). The comparison of δ^15^N values between sizes showed that large tiger sharks (mean 14.1‰) were at a higher trophic level than small sharks (mean 13.1‰) (Fig 2).

**Table 2:** Values of δ13C and δ15N (expressed as ‰; mean ± SD) in the muscle tissue of tiger sharks (*G*. *cuvier*) of different sex and length categories sampled at two locations within the Galapagos Marine Reserve.

Island	Category	Sex	n	Length (cm)	δ^13^C ± SD	δ^15^N ± SD	C/N
**Isabela**	Large	Female	2	351 ± 38.2	–13.7 ± 0.1	14.4 ± 0.6	2.8
		Male	2	284 ± 2.8	–13.2 ± 0.3	14.5 ± 0.6	2.9
	Small	Female	2	191 ± 72.1	–14.2 ± 0.1	13.2 ± 0.4	2.8
		Male	5	192 ± 13.9	–14.2 ± 0.4	13.3 ± 0.2	2.8
**Santa Cruz**	Large	Female	6	363.1 ± 35.2	–13.6 ± 0.3	14.2 ± 0.6	2.8
		Male	4	363.2 ± 37.7	–13.7 ± 0.2	13.6 ± 0.2	2.8
	Small	Female	6	245.6 ± 17.8	–14.1 ± 0.2	12.9 ± 0.3	2.8
		Male	2	240 ± 28.3	–14.1 ± 0.1	12.9 ± 0.1	2.9

### Food sources and isotopic niche

**Fig 3:**
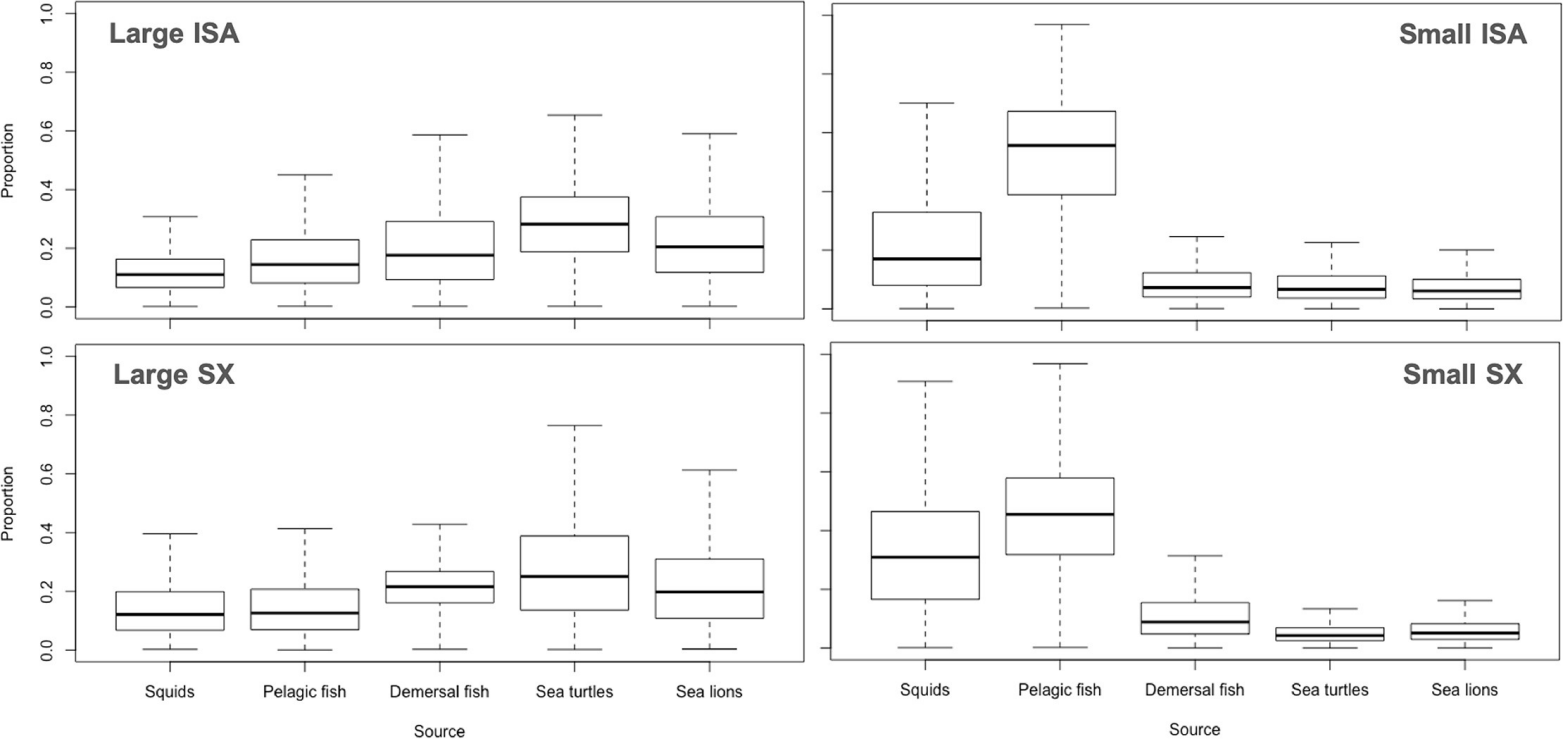
Prey contribution (%) to large and small tiger sharks diet at two locations within the Galapagos Marine Reserve. Box plot produced by the SIMMR model using individual prey isotopic values. Boxes show median value and 95%, 75% and 50% credibility intervals.

We identified five potential prey groups and executed a SIMMR mixing model to account for the relative contribution of each group in the diet. The SIMMR revealed the predominance of green sea turtles (*Chelonia mydas*) in the diet of large tiger sharks at both Isabela and Santa Cruz islands, representing a mean proportion of 28.4% at Isabela (ranging from 15.3% to 41.5%) and 27.1% at Santa Cruz (ranging from 10.8% to 43.4%). Sea lions (*Zalophus wollebaeki*) were the second most dominant item of prey at Isabela (22.3%), while demersal fish (21.8%) and sea lions (21.4%) were the complementary prey at Santa Cruz (Fig 3).

In contrast, pelagic fish were the main prey item on the diet of small tiger sharks both at Isabela and Santa Cruz, with a mean proportion of 51.8% (ranging from 31.1% to 72.5%) and 44.5% (ranging from 25.8% to 63.2%), respectively. Squids were the second most common prey item at Isabela (22.8%), while squids (32.6%) and demersal fish (11.5%) were the second and third most prevalent prey at Santa Cruz (Fig 3). The results of the SIMMR output for all groups of prey and their convergence diagnostics to model fit validate are presented in Table 3.

**Table 3:** Mean proportion, standard deviation (SD) and convergence diagnostics of prey items on the diet of large and small tiger sharks at two locations within the Galapagos Marine Reserve. Model fit is assessed by the Gelman diagnostic, where all parameters must be close to 1. ISA: Isabela Island, SX: Santa Cruz Island.

Size & Island	Sources	Gelman Diagnostics (Point est.)	Contribution (Mean ± SD)	Contribution in Quantiles		
				25%	50%	75%
**Large ISA**	Squid	1	0.12 ± 0.1	0.07	0.11	0.16
	Pelagic fish	1	0.17 ± 0.1	0.08	0.15	0.23
	Demersal fish	1	0.21 ± 0.1	0.09	0.18	0.29
	Sea lions	1	0.22 ± 0.1	0.12	0.21	0.31
	Sea turtles	1	0.28 ± 0.1	0.19	0.28	0.38
**Small ISA**	Squid	1	0.23 ± 0.2	0.08	0.17	0.33
	Pelagic fish	1	0.52 ± 0.2	0.39	0.56	0.67
	Demersal fish	1	0.10 ± 0.1	0.04	0.07	0.12
	Sea lions	1	0.08 ± 0.1	0.03	0.06	0.10
	Sea turtles	1	0.09 ± 0.1	0.04	0.07	0.11
**Large SX**	Squid	1	0.14 ± 0.1	0.07	0.12	0.20
	Pelagic fish	1	0.15 ± 0.1	0.07	0.13	0.21
	Demersal fish	1	0.22 ± 0.1	0.11	0.20	0.27
	Sea lions	1	0.22 ± 0.1	0.14	0.22	0.31
	Sea turtles	1	0.27 ± 0.2	0.16	0.25	0.39
**Small SX**	Squid	1	0.33 ± 0.2	0.17	0.31	0.47
	Pelagic fish	1	0.46 ± 0.2	0.32	0.46	0.58
	Demersal fish	1	0.12 ± 0.1	0.05	0.09	0.16
	Sea lions	1	0.06 ± 0.1	0.03	0.05	0.08
	Sea turtles	1	0.05 ± 0.1	0.03	0.04	0.07

**Fig 4:**
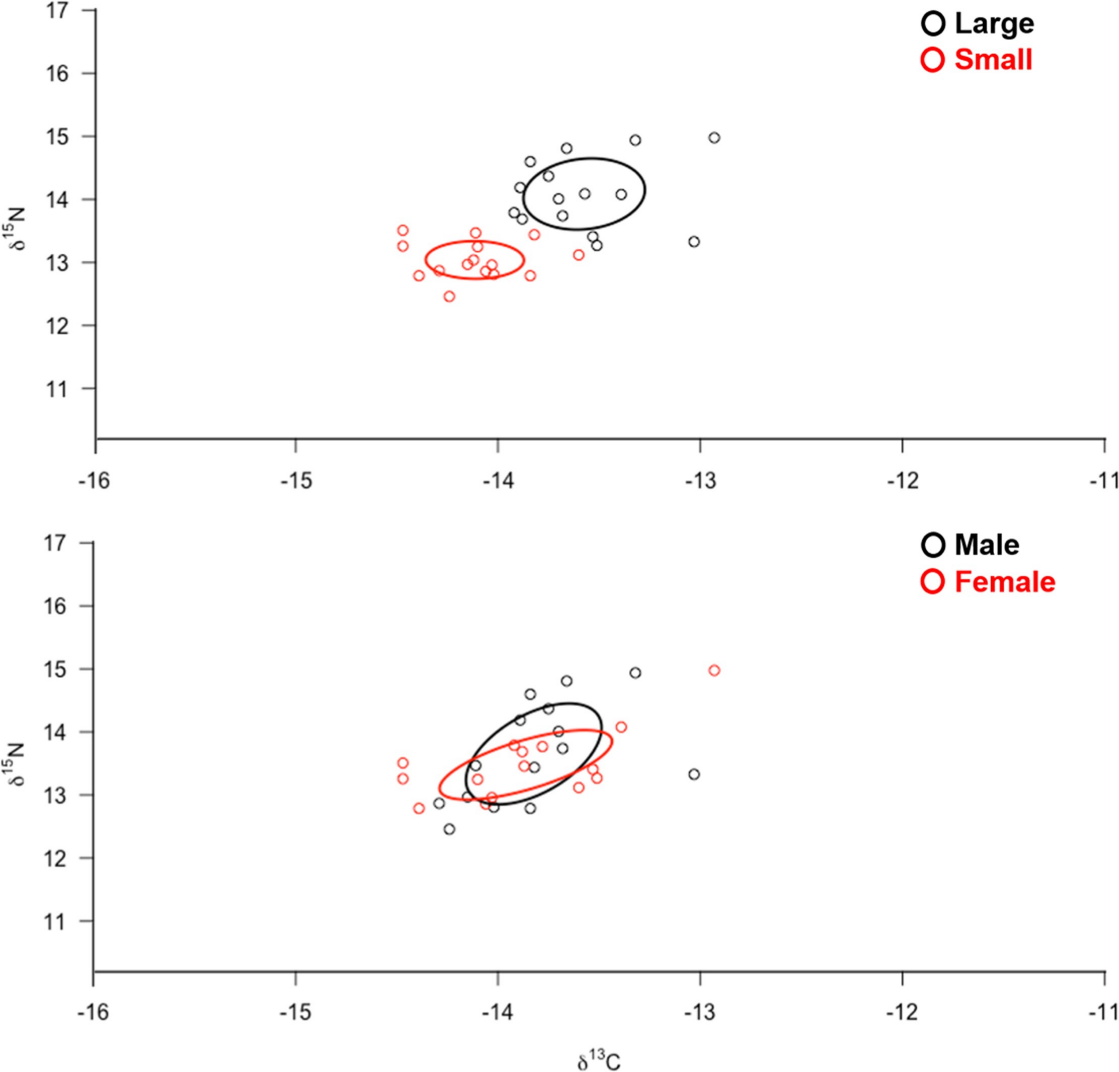
Isotopic niche area (δ^13^C and δ^15^N values) and degree of trophic niche overlap between large vs. small / male vs. female tiger sharks at two locations within the Galapagos Marine Reserve. Values estimated by a convex hull area and the ellipse corrected for the SIBER analysis.

The corrected standard ellipse area (SEAc) in SIBER showed evidence that large and small tiger sharks at Isabela and Santa Cruz islands could be exploiting different types of prey and habitats (Fig 4). The Bayesian ellipse of both groups had a minimal overlap (0.2%), thus confirming different resource use patterns for these groups. Where the large tiger sharks in both islands present a wider isotopic niche compared to the smaller sharks (Fig 4). When accounting for sex, an isotopic overlap was observed between males and females (0.6%) (Fig 4, Table 4). The overlap area of Bayesian ellipses from males and females represented 74.2% of the ellipse surface of the former and 88.7% of the ellipse surface of the latter (Fig 4). The Bayesian ellipses of males were larger and encompassed most of the Bayesian ellipses of females, suggesting a higher diversity of foraging strategies in males compared to females for this species (Fig 4, Table 4).

**Table 4:** Basic Standard Ellipse Area (SEA) and Corrected standard ellipse area (SEAc) measured using Stable Isotope Bayesian Ellipses as an estimate of the trophic niche breadth (TNB) in different sex and length categories of tiger sharks at two locations within the Galapagos Marine Reserve.

Categories	SEA	SEAc	TNB
*G*. *cuvier (Large)*	0.529	0.579	1.341
*G*. *cuvier (Small)*	0.227	0.245	0.585
*G*. *cuvier (Female)*	0.720	0.775	1.748
*G*. *cuvier (Male)*	0.541	0.582	1.307

### Site fidelity around nesting areas

A total of 23 tiger sharks were tagged with satellite transmitters. However, two of the tags failed to provide any data (Table 1). When modelled locations were pooled across the 21 sharks tagged, 91.7% of all points were located within the GMR. Only two of the sharks tagged in 2014 temporarily left the reserve, but both returned to the archipelago during the turtle-nesting season as described in Acuña-Marrero et al. [23].

The estimated core range (CR) area for tiger sharks was 120.95 km^2^ and the activity space (AS) was calculated to cover an area of 1,501.51 km^2^ (Fig 1). Both these areas were located in close proximity to the green turtle nesting beaches of Bachas and Salinas in northern Santa Cruz and Quinta Playa in southern Isabela. In fact, 56.9% of estimated shark locations were within 5 km of these beaches, and as much as 76.9% of the estimated shark locations were within a 10 km buffer.

**Fig 5:**
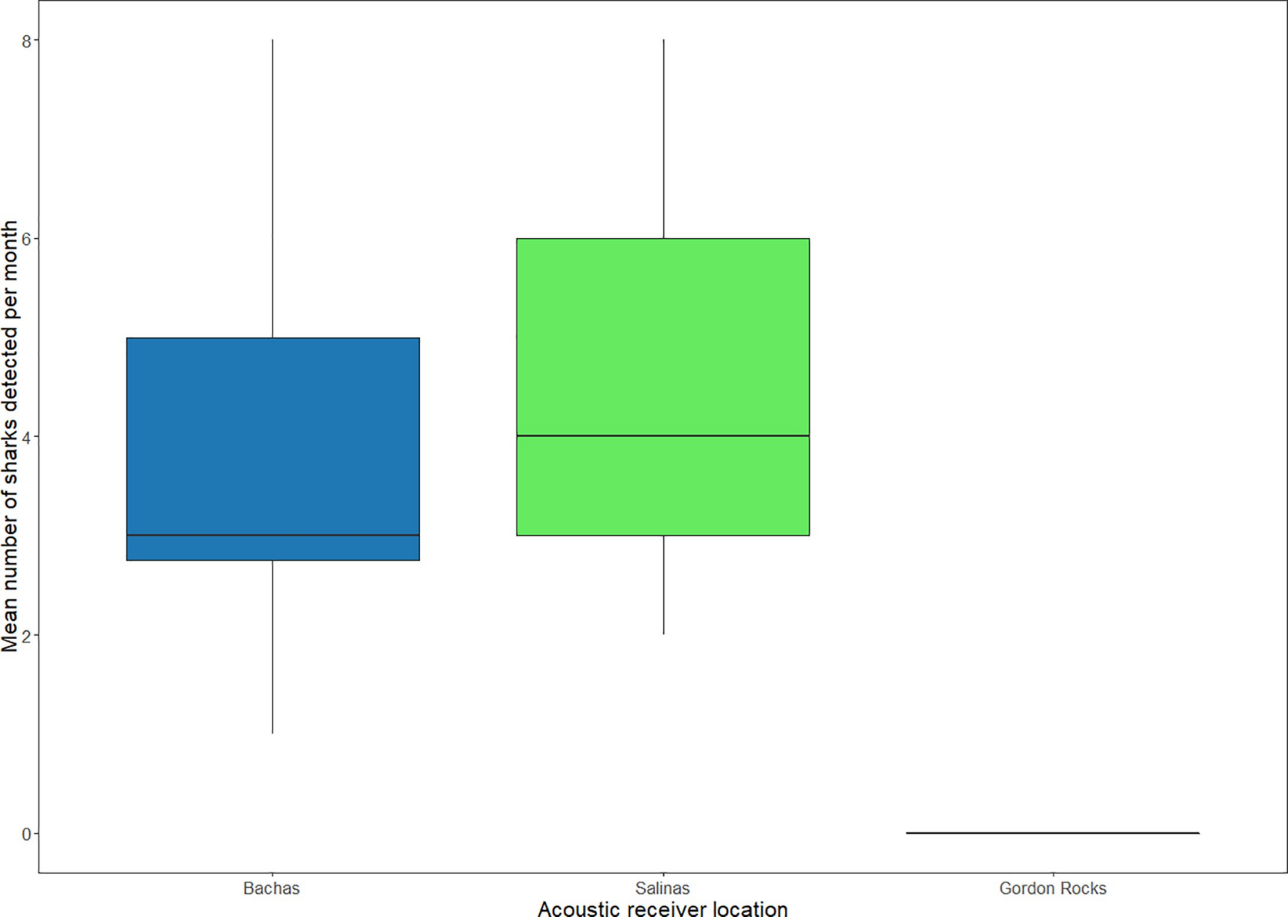
Mean number of tiger sharks (*G*. *cuvier*) detected per month by the underwater acoustic receivers during the study period (October 2014 -November 2017).

**Fig 6:**
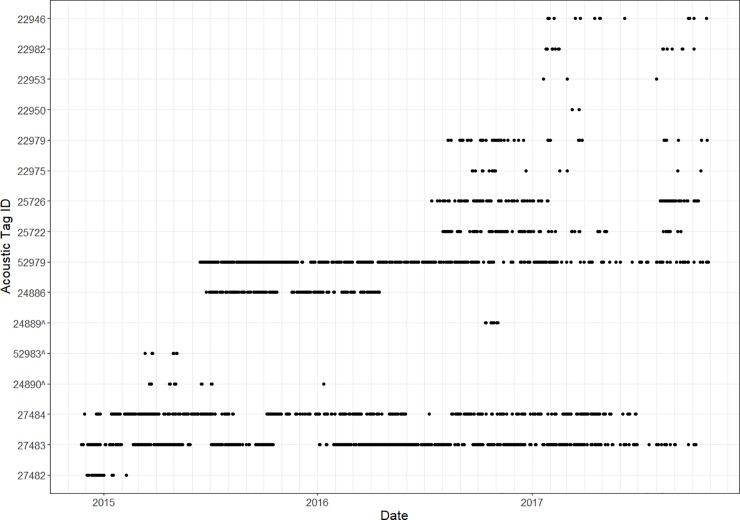
Abacus plot of tiger shark detections. Dots represent dates when individual sharks were detected by underwater acoustic receivers during the study period (October 2014—November 2017). Receivers were located at Salinas and Bachas green sea turtle nesting beaches, Galapagos Islands. ^ denotes sharks tagged at Isabela Island.

Of the 16 tiger sharks fitted with acoustic transmitters around Santa Cruz Island, 13 were detected by the acoustic receivers located on Bachas and Salinas beaches, with both receivers detecting a similar number of sharks per month during the study period (Table 1, Fig 5). However, no sharks were detected at the Gordon Rocks receiver located just 20 km away from the Salinas nesting beach. The three acoustic tags that were not detected by acoustic receivers likely failed, because although not a single acoustic transmission was recorded for these individuals over the three-year study period, transmissions from the satellite tag of one doubled tagged individual were received (GC300114-3). Overall, 76% (n = 10) of working transmitters were regularly detected in the study area for at least 10 months after their capture date (Fig 6). Shark GC130716-2 (25722), a sub-adult female (220 cm TL) tagged in July 2016, was detected within the study area for 14 months until it was captured at an undisclosed location by a fisherman from the Ecuadorian mainland, and its satellite transmitter was returned in October 2017. Without taking into account the three failed acoustic transmitters and the fished individual, 75% of the sharks monitored (n = 9) were still present at the Bachas-Salinas area at the time of the last VR2W receivers download (Table 1, Fig 6).

Only data from Santa Cruz island is presented here given that the acoustic receivers on the south of Isabela island were vandalized on several occasions after the first year of the study in 2015. Three of the sharks tagged around Isabela Island, two sub-adult females and one sub-adult male, were detected for short periods of time during 2015–2016 on the Santa Cruz receivers (Table 1, Fig 6).

## Discussion

This is the first study to investigate the trophic ecology of the tiger shark in the Galapagos Marine Reserve (GMR), and the most comprehensive study to date on this species in the Eastern Pacific. Our results reveal the importance of high trophic level prey (i.e., sea turtles and sea lions) on the diet of adult tiger sharks, and highlight the importance of conducting research within close to pristine marine ecosystems that allow the study of trophic interactions across food webs with limited levels of human interference.

### Spatial dietary patterns

Our stable isotope analysis revealed that green sea turtles (*C*. *mydas*) were the main prey item for large tiger sharks, similarly to what has been previously documented in other locations such as Shark Bay in Western Australia [17] and Raine Island in the Great Barrier Reef [64]. The GMR is one of the most important nesting and foraging sites for the green sea turtle across the Tropical Eastern Pacific, and although most of the nesting occurs during the warm season (December to May), a proportion of the sea turtle population resides within the GMR year-round [32]. This provides a constant and predictable food supply for tiger sharks that results in high levels of residency and a high degree of philopatry within the GMR, with over 90% of total satellite-tracked time across all individuals occurring within the reserve [23].

Our data suggest an isotopic differentiation between Isabela and Santa Cruz green turtle populations within the Galapagos archipelago (Fig 2). In fact, Carrión-Cortez et al. [65] reported significant differences in the foraging behaviour of green sea turtles from Isabela and Santa Cruz islands driven by the presence of the algae *Hypnea* sp., *Caulerpa racermosa* and *Dictyota* sp., which were only present in the diet of sea turtles around Santa Cruz island. This site fidelity could be influenced by the varying oceanographic conditions across the Galapagos and the highly productive upwelling systems in the western region [66]. Therefore, our isotopic and telemetry evidence suggests that tiger sharks within the Galapagos could be segregated into specific island based populations separated by geographical scales of <100 km, since the isotopic contribution in the diet of both populations was represented by the isotopic values present in their main prey item (sea turtles) that inhabit each separate region.

Moreover, based on the satellite transmissions (Fig 1), and considering the limited detections of sharks tagged around Isabela island on the acoustic receivers in Santa Cruz island (Fig 5), it is likely that sharks from each island forage primarily on sea turtles and other resources around their respective islands. The differences in the green sea turtles’ isotopic signatures between Isabela and Santa Cruz could also explain the minimal overlap among large tiger sharks from both islands. Although high mobility has been reported for tiger sharks in other ocean basins, where individuals have migrated over thousands of kilometres [58,67,68], tiger sharks in the Galapagos showed restricted movements within very specific confined geographical areas around the archipelago [23]. This behaviour could be related to the geographic isolation of the Galapagos Islands and the associated limitations to extend their range without depleting energy stores.

The hypothesis of segregated tiger shark populations is supported by a recent archipelago-wide survey using Baited Remote Underwater Video Stations (BRUVS), where the vast majority of the tiger sharks were detected around the central part of the archipelago, specifically to the south and east of Isabela Island and to the north and west of Santa Cruz Island [69]. Moreover, recent genetic evidence for Galapagos shark (*Carcharhinus galapagensis*) populations around the GMR, revealed the existence of two genetically distinct stocks for this highly mobile species: one on the western part of the archipelago (Isabela) and another on the central-eastern part of the archipelago (Santa Cruz and San Cristobal islands) [70]. Genetic studies on tiger sharks could provide further insights on the level of population isolation within the GMR.

### Ontogenetic dietary shifts

Comparison of the diet between large and small tiger sharks within the GMR revealed an ontogenetic shift, with small tiger sharks generally feeding on lower trophic groups. Pelagic fish and squid were the main prey items, similarly to other trophic studies in sub-adult individuals along the coast of Ecuador [22], Western Australia [17] and the north-western Atlantic [21]. Ontogenetic dietary shifts, in which the number and size of prey items increase as tiger sharks increase in size, have also been reported around Hawaii [13], New Caledonia [11], and South Africa [20]. These variations have been suggested to be the result of several factors, including better hunting efficiency in larger sharks, and habitat segregation based on their size [13]. For example, Lea et al. [58] revealed that larger tiger sharks are more migratory and spend more time in areas of high prey biomass, which could enhance foraging opportunities.

Tiger sharks of the Galapagos show a high degree of residency, and both large and small individuals occupy similar geographical areas around the central and western regions of the archipelago [23]. Given that only large sharks feed upon larger prey, such as green sea turtles and sea lions (Fig 3), it is likely that this is a result of increased hunting efficiency with size and/or the use of different foraging habitats. Additional studies using electronic transmitters equipped with pressure sensors, accelerometers or digital cameras [71] could provide further evidence to explain changes in the trophic ecology associated to the size of the tiger shark. However, our results confirmed the top predatory role of tiger sharks at the GMR and further studies should evaluate their importance in maintaining healthy marine ecosystems via top-down control of food webs [2,8,16].

Reports from as early as the 1920s already described frequent tiger shark encounters around the central part of the archipelago, including a description of the predatory event of a ‘monstrous’ tiger shark upon a Galapagos sea lion pup [72,73]. Recent anecdotal evidence from residents and naturalist guides suggests that Galapagos tiger shark populations might be on the rise, although this still needs to be scientifically validated. A sudden increase on the tiger shark sightings around Isla del Coco off the Pacific coast of Costa Rica during the last 10–15 years has been linked to the recurring sighting of a small number of individuals that might have established long-term residency [74]. This increase in tiger shark abundance has been in turn linked to a sharp decline of sightings of mesopredators, such as marble rays (*Taeniurops meyeni*) and white tip reef sharks (*Triaenodon obesus*). Considering the records of neonate and young of the year tiger sharks around the central part of the Galapagos, the presence of suitable nursery and foraging grounds, and the resident behaviour displayed by mature females [23,75], it is likely that the Galapagos tiger shark populations might be on the rise due to self-recruitment. Given the relative pristine environment of the GMR, the Galapagos represents an ideal study site to further investigate predator-prey interactions and their implications for ecosystem dynamics and conservation.
