# Correction: A matter of taste: Spatial and ontogenetic variations on the trophic ecology of the tiger shark at the Galapagos Marine Reserve

**DOI:** 10.1371/journal.pone.0268666

**Published:** 2022-05-12

**Authors:** Pelayo Salinas-de-León, Denisse Fierro-Arcos, Jennifer Suarez-Moncada, Alberto Proaño, Jacob Guachisaca-Salinas, Diego Páez-Rosas

There are errors in the sample sizes reported in Table 1. The correct sample size for the Acoustic tag ID column is n = 25 (28 sharks tagged minus three failed tags). The correct sample size for the Satellite tag ID column is n = 21 (23 sharks tagged minus two failed tags).

The authors provide further information about the source of the satellite and acoustic data summarized in Table 1, as follows:

For 18 sharks captured and fitted with satellite tags between 2014–2015, the satellite data used in this study was obtained from Acuña-Marrero et al. (reference [23] of the article [1]), as indicated in the table caption. For five sharks captured and tagged with satellite tags between 2016–2017, satellite data were newly collected. For 18 sharks captured and tagged with acoustic tags between 2014–2015, the acoustic data used in this study is a combination of re-used data collected 2014–2015 (obtained from Acuña-Marrero et al., reference [23] of the article [1]) and new data detected by the acoustic receiver array September 2015-October 2017. For 10 sharks captured and tagged with acoustic tags between 2016–2017, acoustic data were newly collected.

The authors provide further clarifications about the permits for part of this work. Four tiger sharks captured at Santa Cruz Island, IDs GC300114-1, GC300114-2, GC300114-3, GC300114-4, were initially collected on board OCEARCH research cruise (30 Jan 2014) under Galapagos National Park Directorate (GNPD) Research Permit PC-01-14 issued to Dr. Alex Hearn; Dr. Pelayo Salinas and the Charles Darwin Foundation (CDF) were listed on the permit as a participant and a sponsoring institution, respectively. Subsequent stable isotope analyses of samples from these sharks were carried out under the GNPD permit PC-38-16 issued to Dr. Diego Páez-Rosas. Acoustic data collection September 2015-October 2017 was carried out under GNPD permits issued to CDF; some acoustic data, including on the aforementioned four sharks, were downloaded as publicly available from Acuña-Marrero et al. (reference [23] of the article [1]).
